# Graphene- and Graphene Oxide-Based Nanocomposite Platforms for Electrochemical Biosensing Applications

**DOI:** 10.3390/ijms20122975

**Published:** 2019-06-18

**Authors:** Madasamy Thangamuthu, Kuan Yu Hsieh, Priyank V. Kumar, Guan-Yu Chen

**Affiliations:** 1 Nanophotonics and Metrology Laboratory (NAM), Swiss Federal Institute of Technology Lausanne (EPFL), 1015 Lausanne, Switzerland; madasamy.thangamuthu@epfl.ch; 2 Institute of Biomedical Engineering, College of Electrical and Computer Engineering, National Chiao Tung University, Hsinchu 300, Taiwan; Kuan.Yu@ibm.com; 3 Department of Electrical and Computer Engineering, College of Electrical and Computer Engineering, National Chiao Tung University, Hsinchu 300, Taiwan; 4 School of Chemical Engineering, University of New South Wales, Sydney, NSW 2052, Australia; 5 Department of Biological Science and Technology, College of Biological Science and Technology, National Chiao Tung University, Hsinchu 300, Taiwan

**Keywords:** graphene, graphene oxide, reduced graphene oxide, electrochemical biosensor, cell capture

## Abstract

Graphene and its derivatives such as graphene oxide (GO) and reduced GO (rGO) offer excellent electrical, mechanical and electrochemical properties. Further, due to the presence of high surface area, and a rich oxygen and defect framework, they are able to form nanocomposites with metal/semiconductor nanoparticles, metal oxides, quantum dots and polymers. Such nanocomposites are becoming increasingly useful as electrochemical biosensing platforms. In this review, we present a brief introduction on the aforementioned graphene derivatives, and discuss their synthetic strategies and structure–property relationships important for biosensing. We then highlight different nanocomposite platforms that have been developed for electrochemical biosensing, introducing enzymatic biosensors, followed by non-enzymatic biosensors and immunosensors. Additionally, we briefly discuss their role in the emerging field of biomedical cell capture. Finally, a brief outlook on these topics is presented.

## 1. Introduction

### 1.1. Graphene and Its Derivatives: Synthesis and Properties

**Figure 1:**
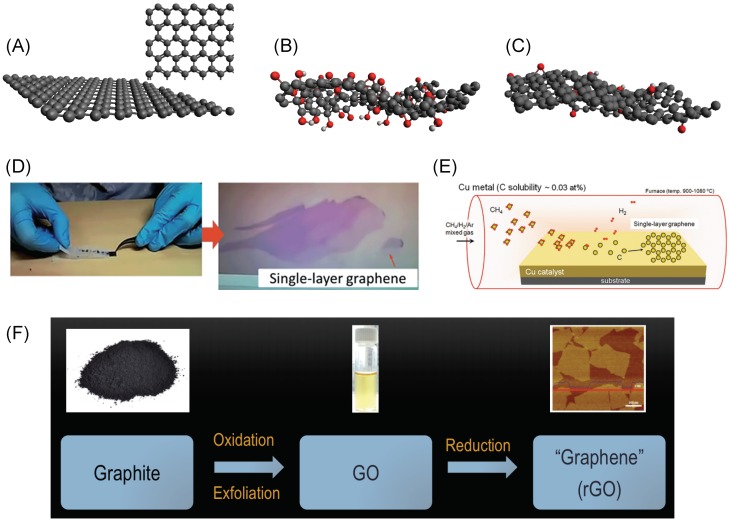
(**A**) The hexagonal honeycomb structure of graphene. (**B**) The amorphous structures of graphene oxide and (**C**) reduced graphene oxide. Black, red, and white spheres represent carbon, oxygen, and hydrogen atoms, respectively. Schematics of the synthesis of graphene monolayers using (**D**) the mechanical exfoliation method, (**E**) chemical vapor deposition method, and (**F**) the solution-based method. Figures reproduced with permission from: (**D**) [10], RSC^©^; (**E**) [11], Springer Japan^©^.

Graphene is a one-atom thick form of carbon, where the carbon atoms are arranged regularly in a hexagonal lattice (Figure 1A) [1]. Because of a unique combination of excellent electrical, optical, chemical and mechanical properties, it has impacted technologies ranging from electronic, optoelectronic [2,3] and biomedical applications [4] at the nanoscale to membrane [5,6] and mechanical applications [7] at the macroscale [8]. As such, groups have constantly researched different ways to obtain graphene monolayers from graphite, its bulk counterpart.

To produce high-quality graphene monolayers that exhibit excellent sheet properties, the mechanical exfoliation and the chemical vapor deposition (CVD) methods have remained popular [9,10,11]. In mechanical exfoliation, an adhesive such as a scotch-tape is employed to peel atomically-thin layers of graphene from graphite (Figure 1D) [10], while, in CVD, a carbon source is typically used as a precursor in a high-temperature reaction chamber to grow graphene on a suitable substrate such as copper (Figure 1E) [11,12,13]. These methods yield graphene that can be employed in high-quality electronic and optoelectronic devices. For instance, electronic mobility values of 10${}^{5}$ cm${}^{2}$V${}^{-1}$s${}^{-1}$ can be reached in CVD-grown graphene [14]. In contrast, silicon, a commonly used semiconductor, exhibits an electronic mobility value of about 1000 cm${}^{2}$V${}^{-1}$s${}^{-1}$. The intrinsic tensile strength of CVD-graphene has been measured to be 118 GPa, a value greater than that of steel at the nanoscale, a commonly used structural material [15].

In 2006, Ruoff and co-workers employed a solution-based approach to produce graphene monolayers and thin-films on a large scale, which is termed as the chemical exfoliation method (Figure 1F) [16,17]. In this approach, graphite is oxidized following a redox reaction forming graphite oxide [18]. Due to weakened van der Waals forces between the individual sheets, graphite oxide can be readily exfoliated in a solvent such as water, yielding single layers of oxidized graphene called graphene oxide (GO), as shown in Figure 1B. Deposition of such monolayers on to suitable substrates or electrodes is easily achieved through spin coating or drop casting methods [19].

Traditionally, since the key interest is to obtain large-area, single sheets of high electrical quality graphene with no defects or oxygens, large efforts have been put into studying the process of reduction of GO, i.e., removal of oxygen atoms from the basal plane. Generally, researchers have used two methods to achieve this goal: (1) Thermal reduction is where high temperatures are utilized to break C-O bonds and thus remove oxygen groups [20,21,22,23,24,25,26,27,28]. Typically, GO thin films are deposited on a substrate are held at temperatures >150 ${}^{\circ }$C in air or vacuum chambers for 15–60 min to remove oxygen atoms [23]. (2) Chemical reduction uses reduction agents such as hydrazine (N${}_{2}$H${}_{4}$), sodium borohydride (NaBH${}_{4}$), ascorbic acid, and hydrohalic acids [2,29,30]. In addition to these, electrochemical [31] and microwave reduction of GO films [32] have also been employed. Although most of the oxygen is removed following these reduction protocols, residual oxygen on the order of a few atomic percent remains in these graphene sheets, as shown in Figure 1C. Hence, these structures are termed as reduced graphene oxide (rGO). It is also common to simply use the term graphene for rGO.

Since the scalability and solution-processability allows for facile fabrication of GO- and rGO-based electrodes, they have been widely employed to design electrochemical sensors [33]. Further, excellent electrical conductivity, redox properties, stability, and the presence of oxygen functional groups and defects in rGO thin films [34] enhance their electrochemical performance and their ability to bind biomolecules [35].

### 1.2. GO and rGO: Structure–Property Relationships

GO is known to contain several oxygen functional groups (epoxy and hydroxyl primarily) that are bonded to the underlying graphene plane. In addition, the sheet edges are known to be functionalized with carboxyls and lactols [36]. GO is thus known to contain a mixture of *sp*${}^{2}$- and *sp*${}^{3}$-hybridized carbon atoms. Further, GO is amorphous in nature, meaning that the oxygen functional groups are randomly attached on to the graphene plane [34,37,38]. Typically, GO is characterized by its oxygen concentration, which is in the range 30–35 atomic percent (at%) following the Hummers’ synthesis protocol [18]. It is also characterized by the ratio of epoxy to hydroxyl functional groups present in GO. A range of values between 0.7 and 1.3 is generally observed for this ratio [20].

**Figure 2:**
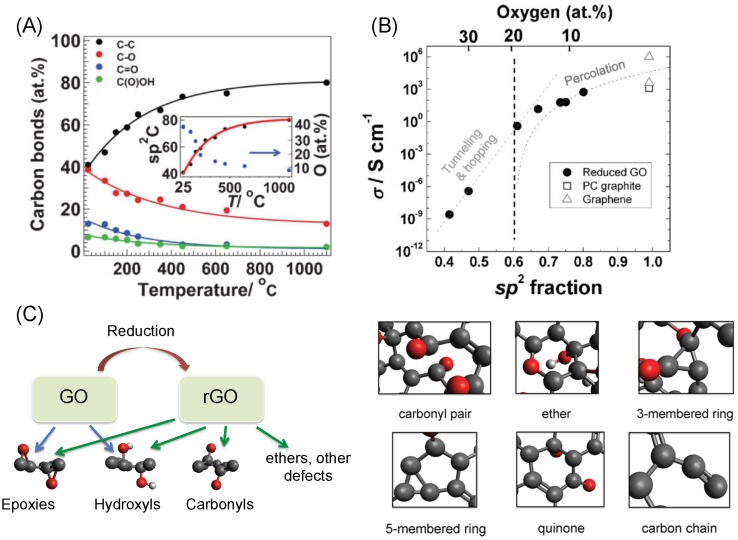
(**A**) Evolution of *sp*${}^{2}$ C=C carbon and different oxygen functionalities at different annealing temperatures. The inset correlates the total oxygen concentration with the *sp*${}^{2}$ C=C content at different reduction temperatures. (**B**) Conductivity of rGO thin films as a function of the *sp*${}^{2}$ C=C content/oxygen content. (**C**) Dominant oxygen functional groups present in GO and rGO, along with close-ups of various other oxygen groups and defects that are created upon reduction of GO. Figures reproduced with permission from: (**A**,**B**) [23], Wiley^©^.

The reduction of GO leads to many changes in the sheet’s chemical structure, which in turn provides opportunities for tailoring its properties. Freshly prepared GO is an insulator with a sheet resistance of about 9 GΩ/sq. since it contains a large fraction of *sp*${}^{3}$-hybridized carbon atoms and isolated *sp*${}^{2}$-hybridized carbon-atom sites [27,39]. Upon reduction, GO undergoes an insulator–semiconductor–semimetal transition due to continuous removal of oxygen atoms, leading to restoration of *sp*${}^{2}$-hybridized carbon atoms (Figure 2A) [23,40]. Thus, the carrier mobility can be tuned over up to 12 orders of magnitude by monitoring the oxygen content (and the fraction of *sp*${}^{2}$- to *sp*${}^{3}$-hybridized carbon atoms), as shown in Figure 2B [23]. Although we note that the presence of residual oxygen in rGO limits the carrier mobilities from reaching the extraordinary values of the mobilities obtained in mechanically-exfoliated or CVD graphene [20,23], high electron and hole mobilities in the range 100–1000 cm${}^{2}$V${}^{-1}$s${}^{-1}$ and sometimes exceeding 1000 cm${}^{2}$V${}^{-1}$s${}^{-1}$ have been measured in rGO thin films [32]. These properties allow for superior charge transfer characteristics at rGO-based electrodes leading to larger currents and better signal-to-noise ratios in sensing applications [41].

Experiments and theory have shown the formation of CO and CO${}_{2}$ molecules during the reduction process [24,25,26]. This means that the carbon atoms are also removed from the basal plane, introducing various defect structures. These include carbonyls, ethers, carbon chains and vacancies (induced by carbon removal) along with the usual oxygen-containing groups such as the epoxies and the hydroxyls (Figure 2C) [20,34]. These oxygen and defect sites could allow for better binding of biomolecules, support efficient electrical wiring of the redox centers of the metalloproteins to the electrode, and improve specificity when multiple analytes are present. Since such defects lead to the higher density of electronic states at the Fermi level, they also help reduce the overpotential associated with biosensing [41,42].

Another important property of 2D graphene films is their large surface area, which can help load more biomolecules onto the surface [43,44]. Furthermore, they can act as effective electron mediators, which fastens the electron transfer between the metal active site of the biomolecules and the electrode surface [45]. Other important properties include transparency, cost-effectiveness, wide electrochemical potential windows, low electrical resistance compared to glassy carbon electrodes and well-defined redox peaks [46,47]. Overall, GO and its derivatives form a suitable material set for miniaturizing and improving the efficiency of biosensing devices.

## 2. Graphene/Graphene Oxide Nanomaterials Based Electrochemical Biosensors

### 2.1. Enzymatic Biosensor

**Figure 3:**
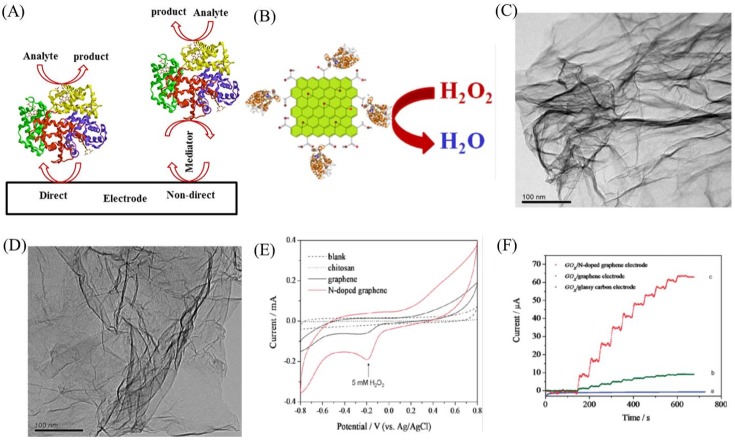
(**A**) Schematic of the direct and mediated electrochemical biosensor. (**B**) Detection of H${}_{2}$O${}_{2}$ on graphene quantum dots modified electrode. (**C**,**D**) TEM images of the graphene and N-doped graphene. (**E**) H${}_{2}$O${}_{2}$ detection on graphene and N-doped graphene. (**F**) Glucose detection on graphene and N-doped graphene modified electrodes. Figures reproduced with permission from: (**B**) [54], Springer^©^; and (**C**–**F**) [55], ACS^©^.

Detection and determination of biomolecules are clinically highly significant for diagnosis and treatment of various diseases. Enzymatic biosensor is a very well accepted system for sensing biomolecules based on their electrochemical reaction (oxidation or reduction) with the enzyme, which is immobilized on the electrode surface [48,49,50,51]. The electrochemical output signal corresponds to the concentration of the analyte molecule. The analytical performance of such a biosensor mainly depends on the electron transfer between the metal active site of the enzyme and the electrode surface. Figure 3A shows a schematic of enzymatic biosensor. Enzyme can be directly immobilized on the electrode surface to achieve direct electron transfer between the electrode and the enzyme. However, it might result in the denaturation of the enzyme and hence affect the biosensor response. To improve enzyme adsorption, improve stability and enhance the direct electron transfer, nanomaterials have been widely used as immobilization matrix, a mediator between the enzyme and the electrode [52]. As explained in the Introduction, graphene and its oxidized derivative-based nanomaterials show excellent electrochemical properties, viz. high electrical conductivity, access to defect sites, large surface area, better electrocatalytic activity and excellent electron transfer rates, which are promising for fabricating enzymatic biosensors [53]. The oxygen functional groups of the GO and rGO are hydrophilic giving opportunities to integrate them with metal nanoparticles, metal oxides, semiconducting nanoparticles, quantum dots and polymers to improve the electrochemical biosensor performances. Furthermore, nanocomposites made up of graphene-based nanomaterials are also very useful for sensing application. Electrochemical biosensors fabricated using graphene nanomaterials are also cost effective compared to the conventional gold and platinum electrodes. Several graphene-based nanomaterials/nanocomposites have been reported for the detection and determination of biomolecules such as hydrogen peroxide (H${}_{2}$O${}_{2}$), glucose, nicotinamide adenine dinucleotide (NADH), deoxyribonucleic acid (DNA), urea, cholesterol, etc. In the following paragraphs, we discuss a few of them.

H${}_{2}$O${}_{2}$ is an essential mediator in biological processes and hence its measurement is highly imperative for biomedical applications [56]. Although several electrochemical biosensors have been reported the detection of H${}_{2}$O${}_{2}$, advancement to reduce the oxidation/reduction overpotential is still a hot topic in this field. Detection of H${}_{2}$O${}_{2}$ at lower potential avoids interference from other species. Graphene has been shown to achieve this by enhancing the electron transfer rate [57]. Recently, Zou et al. showed the detection of H${}_{2}$O${}_{2}$ by immobilizing horseradish peroxidase (HRP) on rGO using aminopyrene (AP) as a linker. The covalent bond between AP and HRP, and the *π*–*π* interactions between AP and rGO sheets resulted in improved electron transfer across the nanocomposite, enabling suitable detection of H${}_{2}$O${}_{2}$ [58]. Other nanocomposites involving rGO nanosheets have been reported in Refs. [59,60], for example. More examples along with the performance of various graphene-based enzymatic biosensors are highlighted in Table 1.

In addition to rGO, other graphene derivatives have also been employed. Zhou et al. used graphene electrodes to immobilize sarcosineoxidase for the detection of H${}_{2}$O${}_{2}$ in blood serum samples [61]. Muthurasu et al. (Figure 3B) used graphene quantum dots (GQD) modified electrode, as a suitable matrix, for HRP immobilization and observed excellent electrochemical performance towards H${}_{2}$O${}_{2}$ detection [54]. Shao et al. studied pure graphene and nitrogen-doped graphene (N-doped graphene) electrodes for H${}_{2}$O${}_{2}$ reduction and found that N-doped graphene performed much better than the pure one [62]. Wang et al. observed the same effect on N-doped graphene for H${}_{2}$O${}_{2}$ detection. Figure 3C,D shows transmission electron microscopy (TEM) images of pure and N-doped graphene [55]. Figure 3E clearly illustrates that the N-doped graphene shows much better electrochemical performance towards H${}_{2}$O${}_{2}$ detection and the reason is due to the presence of nitrogen functional groups, oxygen-containing groups and structural defects.

Graphene derivatives offer suitable matrix for immobilization of oxidase enzymes and hence are ideal for glucose detection. There are several reports on glucose oxidase (GOx) immobilized graphene electrodes for glucose detection [63,64,65,66]. It has been shown that rGO-based glucose biosensor is better than the GO-based sensor due to higher conductivity of rGO [67]. As discussed above, the obtained higher conductivity is due to the conjugated graphitic network revival after reduction. It is further evidenced by the electrochemical impedance spectra that rGO shows less charge transfer resistance compared to GO. In addition, rGO-metal oxide nanocomposites have been reported for glucose detection [68,69,70]. For instance, Dey et al. showed a one-pot synthesis of rGO-ZnO electrodes by reducing GO directly with zinc, and then immobilized GOx onto the fabricated nanocomposites [68].

Other than rGO, Kang et al. reported a graphene–chitosan nanocomposite matrix to immobilize GOx for glucose detection with wider linear range of detection, lower detection limit and much higher sensitivity [71]. Shan et al. studied a negatively charged GOx adsorption on positively charged polyvinylpyrrolidone (PVP) protected graphene electrode for glucose detection [72]. Wang et al. used N-doped graphene for glucose detection. As shown in Figure 3F, it is obvious that N-doped graphene electrode shows superior performance compared to other electrodes [55]. Similarly, GQD was also reported for highly sensitive glucose detection [73]. The obtained higher sensitivity was due to the abundant hydrophilic edges and planes of graphene in addition to the large-surface to volume ratio.

**Figure 4:**
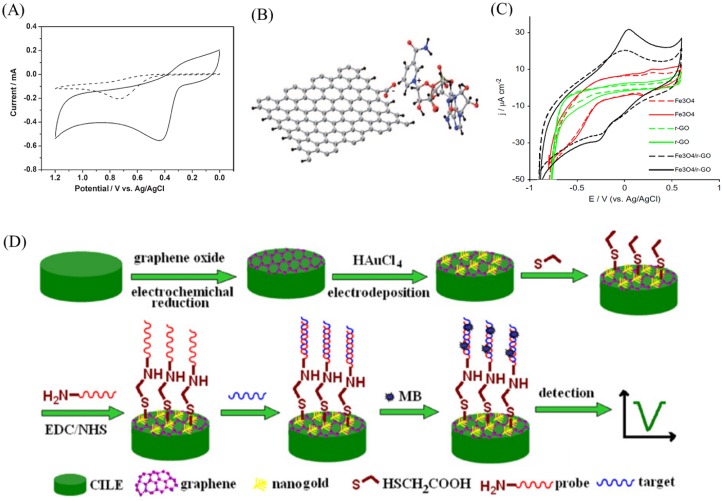
(**A**) Cyclic voltammetric responses of the bare GC (dashed line) and rGO-based GC (solid line) in 0.1 M PBS (pH 6.8) PBS containing 1 mM NADH. (**B**) Graphene edge terminated by hydrogen atoms and containing one -COO- group. (**C**) Electrochemical responses of Fe${}_{3}$O${}_{4}$/GC, r-GO/GC and Fe${}_{3}$O${}_{4}$/r-GO/GC electrodes in 0.1 M PBS (pH 7.0) and scan rate 20 mV s${}^{-1}$ in the absence (dashed line) and presence (solid line) of 0.5 mM NADH. (**D**) Schematic representation of the electrochemical DNA biosensor fabrication and detection. Figures reproduced with permission from: (**A**) [75], Wiley^©^; (**B**), [76], Wiley^©^; and (**C**) [77], Elsevier^©^; and (**D**) [78], Elsevier^©^.

NADH is a redox carrier in metabolic processes and a coenzyme in all living cells. It participates in several hundred enzymatic reactions and hence the detection of NADH is of paramount importance [74]. The oxidation of NADH at large over potential and the accumulation of reaction products are the real challenges for the electrochemical detection of NADH. Graphene-based electrochemical biosensors can overcome these limitations by shifting the oxidation potential of NADH. For instance, Tang et al. studied the electrochemical oxidation of NADH on chemically reduced GO (CrGO) electrode, as shown in Figure 4A. The glassy carbon electrode (dashed line) shows the oxidation of NADH at 0.75 V *vs*. Ag/AgCl whereas CrGO modified glassy carbon (solid line) electrode reduced the potential to 0.42 V which is about 300 mV less for NADH oxidation [75].

The high density of edge-plane-like defective sites on CrGO offering many active sites for electron transfer to NADH was the reason for oxidation of NADH at low potential. Similarly, Liu et al. reported the NADH oxidation at lower potential by functionalizing graphene with methylene green [79]. Pumera et al. claimed that the oxygen functional groups at the graphene edges and edge-like defects supported the NAD+/NADH adsorption [76]. Figure 4B shows the adsorption of NADH on graphene edge terminated by hydrogen atoms and one carboxyl group. In the absence of the carbonyl group, passivation effect was observed, confirming that the oxygen-containing groups play a crucial role for biomolecule adsorption. Teymourian et al. reported Fe${}_{3}$O${}_{4}$ magnetic nanoparticle-loaded rGO nanosheets for NADH oxidation at low potential, 0.05 V [77]. Figure 4C shows that the composite of magnetic nanoparticles and rGO exhibits better electrochemical activity towards NADH. In a study, Gasnier et al. prepared graphene paste electrodes by mixing graphite, mineral oil and rGO. They then studied its electrochemical performance towards NAHD [80].

The application of graphene nanomaterial-based electrodes has also been extended to DNA detection. Sun et al. developed an electrochemical DNA biosensor for the detection of Listeria monocytogenes hly single-strand DNA (ssDNA) sequences using a gold nanoparticle and electrochemically reduced graphene oxide (ErGO) nanocomposite [78]. A schematic of the DNA sensor is shown in Figure 4D, explaining the preparation and the DNA detection mechanism. The linear detection range was 1 pM to 1 μM with a detection limit of 0.3 pM. Zhou et al. developed a label-free electrochemical DNA biosensor for the detection of four different bases in both ssDNA and dsDNA using CrGO [81]. Jia et al. reported a highly sensitive, light addressable potentiometric sensor using GO to probe ssDNA with a detection limit of 1 pM [82].

Detection and monitoring of cholesterol is important to prevent the formation of atherosclerotic lesions in the coronary arteries for persons who have high serum cholesterol levels [83]. Pramanik et al. recently reported a polypyrrole (PPy)–rGO–cholesterol oxidase (ChOx) biocomposite for cholesterol detection. They realized a one-step biosensor fabrication protocol by co-depositing rGO and ChOx during electropoymerization of pyrrole and demonstrated enhanced cholesterol sensing enabled by direct electron transfer [84]. Another example of cholesterol detection by forming rGo-Pd nanocomposites is reported in Ref. [85].

**Figure 5:**
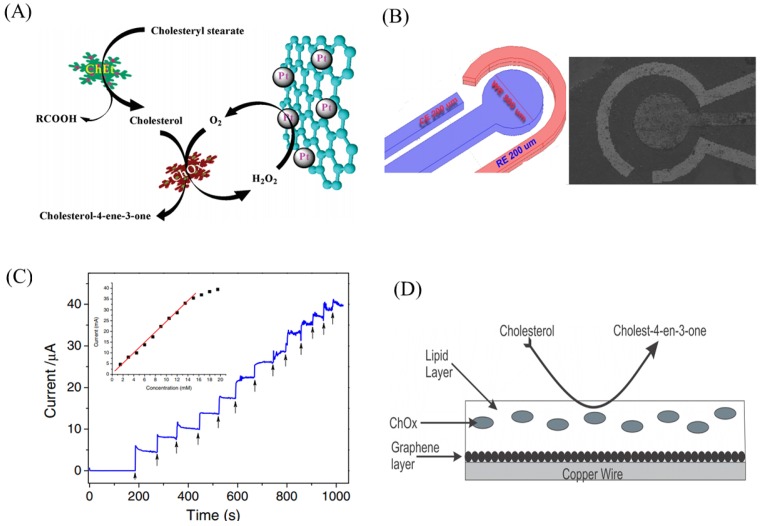
(**A**) Schematic of the the GNS-nPt-Based Biosensor for cholesterol detection. (**B**) A schematic and SEM image of the graphene electrode modified with Fe${}_{3}$O${}_{4}$-doped polyaniline film. (**C**) Amperometric responses obtained for different added cholesterol concentrations (inset: the calibration curve of the cholesterol sensor). (**D**) Schematic of the potentiometric sensor design. Figures reproduced with permission from: (**A**) [86], ACS^©^; (**B**,**C**) [87], IOP Publishing^©^; and (**D**) [88], Gruyter GmbH^©^.

Additionally, Dey et al. reported a bienzymatic electrode i.e., ChOx and cholesterol esterase immobilized on graphene and platinum nanoparticle hybrid for cholesterol detection [86].This bienzymatic electrode was very sensitive and selective to cholesterol with fast response time. The same electrode configuration was fabricated on screen printed electrode to reduce the measurement volume. Figure 5A shows a schematic of the bienzymatic electrode for cholesterol detection. Nguyen et al. immobilized ChOx on CVD synthesized graphene electrode modified with Fe${}_{3}$O${}_{4}$-doped polyaniline film for cholesterol detection [87]. Figure 5B,C shows the electrode design and the amperometric responses for different concentrations of cholesterol. A novel potentiometric sensor for cholesterol was reported by Nikoleli et al., in which a stable polymeric lipid membrane mixed with ChOx on graphene is used [88]. Figure 5D shows a schematic of the sensor electrode and the detection mechanism. The strong biocompatibility was achieved using lipid membrane and hence recommended for real biological and blood sample detection.

### 2.2. Non-Enzymatic Biosensor

**Figure 6:**
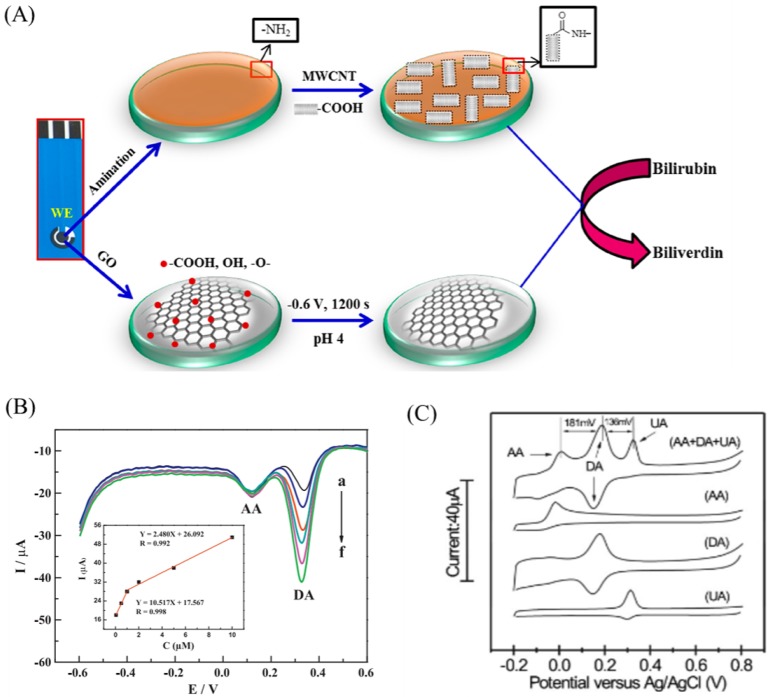
(**A**) Schematic showing the preparation of the MWCNT based (top row) or electrochemically reduced graphene oxide (ErGO) based (bottom row) bilirubin sensors. (**B**) Differential pulse voltammograms of DA (from a to f: 0.05, 0.5, 1, 2, 5 and 10 μM) at Gr/p-ABA/GCE in the presence of 100 μM AA. Inset: calibration plots of the oxidation peak current versus different concentrations of DA. (**C**) CV profiles of the MGNF electrodes in the solution of 50 mM, pH 7.0 PBS with 1 mM AA, 0.1 mM DA, and 0.1 mM UA. Figures reproduced with permission from: (**A**) [42], MDPI^©^; (**B**) [104], Elsevier^©^; and (**C**) [105], Wiley Interscience^©^.

Electrochemical detection of clinically important biomarkers using non-enzymatic electrodes fabricated using graphene-based nanomaterials is an another important application. Thangamuthu et al. developed a non-enzymatic sensor for bilirubin detection, an important biomarker of jaundice, using ErGO [42]. In a screen-printed carbon electrode, ErGO was functionalized and its electrochemical activity towards bilirubin was comparatively studied with MWCNT functionalized electrode. They found that the ErGO electrode performed much better than the MWCNT electrode in terms of detection limit, sensitivity, and range of detection. It is attributed to the faster electron transfer rate and higher electrical conductivity of ErGO. Further, the selectivity was ensured by using nafion membrane coating. Figure 6A shows a schematic of the non-enzymatic bilirubin sensor developed using ErGO and MWCNT. It offers a low-cost, reliable, and miniaturized point-of-care electrochemical sensor for bilirubin. Similarly, graphene nanomaterials based non-enzymatic electrodes were reported for the detection of dopamine, ascorbic acid (AA) and uric acid (UA). Detection of dopamine in the presence of AA is challenging due to the overlapping of electrochemical oxidation potential. Huang et al. reported a selective determination of dopamine in the presence of AA using graphene/p-aminobenzoic acid composite film [104]. The attractive interaction of dopamine cations with the negatively charged nanocomposite film makes the selective detection of dopamine using differential pulse voltammetry possible. The electrochemical oxidation peaks for dopamine and AA were well separated up to 220 mV (Figure 6B). In a study, Shang et al. developed a non-enzymatic sensor for the simultaneous detection of dopamine, AA and UA using multilayer graphene nanoflake films (MGNFs) [105]. They found that the edge planes/defects essentially enhance the electron-transfer rate and active electrocatalytic properties. It is clearly shown in Figure 6C that the MGNFs electrodes successfully detected dopamine, AA and UA simultaneously. Zhang et al. reported a nanocomposite made up of cuprous oxide and graphene for the electrochemical oxidation of dopamine [106].

In addition to detection of aforementioned biomarkers, graphene-, GO- and rGO-based non-enzymatic electrodes have been constructed for the detection of glucose, H${}_{2}$O${}_{2}$ and cholesterol. These are summarized in Table 2.

### 2.3. Immunosensor

Graphene nanomaterial-based electrodes have also been used to develop electrochemical immunosensors. The specific interaction between the antigen and antibody confirms the high selectivity and sensitivity of the immunosensor. Electrochemical immunoassays are well known for their simplicity, high sensitivity and selectivity, large-scale manufacturability, volume miniaturization, and rapid analysis. It can be a sandwich type sensor or a label-free type immunosensor. Several electrochemical immunosensors were reported using graphene nanomaterials for the detection of well-known biomarkers such as carcinoembryonic antigen (CEA), Interleukin-6 (IL-6), human chorionic gonadotropin (hCG), and prostate specific antigen (PSA).

Saeed et al. fabricated DNA-modified (ERBB2c and CD24c) Au–GO nanocomposites for the early detection of breast cancer markers. A sandwich-type sensor strategy was employed here and the sensitive detection of ERBB2 and CD24 [123]. In addition, by using CdSe quantum dot-functionalized polystyrene microscpheres as a bioprobe along with GO–polyaniline (PANI) nanocomposites, Wang et al. reported ultrasensitive detection of tumor cells [124]. They demonstrated a detection limit of 3 cells/ML and attributed this performance to high electron transfer rate and tumor cell loading on the nanocomposites. Table 3 summarizes the performance of additional immunosensors based on graphene nanomaterials.

**Figure 7:**
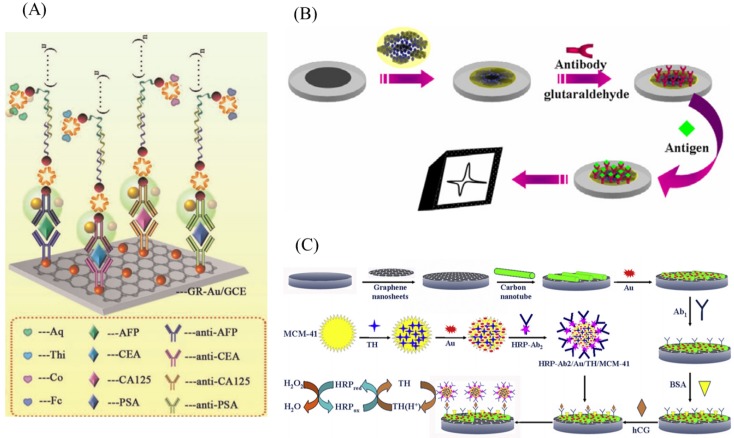
(**A**) Schematic illustration of the principle of sandwich-type simultaneous detection of four antigens. (**B**) Fabrication steps of the label-free electrochemical immunosensor. (**C**) Fabrication process of Au/TH/MCM-41 nanomaterials and the measurement protocol of the electrochemical immunosensor. Figures reproduced with permission from: (**A**) [126], Elsevier^©^; (**B**) [127], Elsevier^©^; and(**C**) [129], Elsevier^©^.

In another example, Huang et al. reported a silver and gold nanoparticle-coated graphene sandwich electrode for the detection of tumor cells - CEA antigen [125]. This sandwich immunosensor was able to detect the CEA antigen with a linear range of 10 to 1.2 × 10${}^{5}$ pg·mL${}^{-1}$ and a detection limit of 8 pg·mL${}^{-1}$. Zhu et al. developed a sandwich type immunosensor for the ultrasensitive simultaneous detection of four antigens using graphene–gold hybrid film [126]. Figure 7A shows a schematic of the sandwich immunosensor electrode in which four different antibodies were immobilized that are specific to their antigens. Mao et al. used label-free electrochemical immunosensor to detect PSA using graphene sheet–methylene blue–chitosan nanocomposite [127]. Figure 7B shows a schematic of the label-free electrochemical immunosensor fabrication. The nanocomposite matrix showed higher binding affinity towards antibody of PSA. This label-free immunosensor was able to detect a minimum concentration of 13 pg·mL${}^{-1}$ of PSA. It was then finally applied to measure PSA in serum samples. To increase the sensitivity of the immunosensor, other nanomaterials such as QD, CNTs, and metal NPs combined nanocomposites were also reported. For example, QD-functionalized graphene sheets were used to develop sandwich type immunosensors for the detection of PSA [128] and used to detect the PSA in patient serum samples, thus making them suitable for clinical analysis. Similarly, Lu et al. developed an immunosensor for the detection of hCG using gold nanoparticles-MWCNT-graphene composite electrode [129]. Figure 7C shows a step-by-step fabrication and detection protocol involved in an hCG immunosensor.

## 3. Graphene/Graphene Oxide Materials for Biomedical Cell Capture and Other Biomedical Applications

It is clear that GO presents a unique 2D surface that includes both *sp*${}^{2}$ (crystalline) and *sp*${}^{3}$ (oxygen groups, defects, etc.) domains. While the rich oxygen framework has not only enhanced GO’s ability to bind biological molecules, the confined graphitic regions have enabled fluorescence and quenching, making GO promising in biomedical applications [138,139,140,141,142,143]. These derivatives have been used for designing advanced functional biological sensing platforms, including affinity-based biosensor [144], fluorescence resonance energy transfer (FRET)-based biosensors [145], surface-enhanced Raman spectroscopy (SERS) [146], electrochemical detection [147] and laser desorption/ionization mass spectrometry (LDI-MS) [148,149].

**Figure 8:**
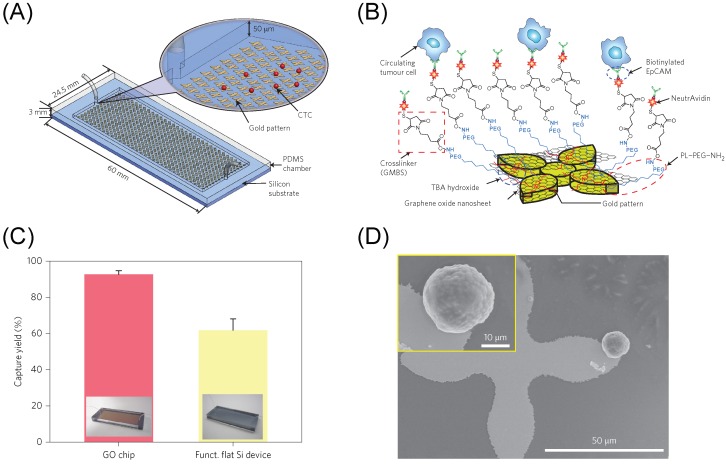
(**A**) Schematic diagram of the GO chip used for capturing circulating tumor cells. (**B**) Schematic showing the conjugation chemistry between functionalized GO nanosheets and EpCAM antibodies. GO nanosheets are adsorbed onto the gold pattern. The GMBS crosslinker binds to PL-PEG-NH2 onto the GO nanosheets. The NeutrAvidin is connected to the GMBS and biotinylated EpCAM. (**C**) Cell capture efficiency values of functionalized GO and flat silicon chips. (**D**) SEM image of gold patterns showing a capture tumor cell. Inset: magnified SEM image of the captured cell. Figures reproduced with permission from: (**A**–**D**) [150], NPG^©^.

Some researchers have started to use GO as an interface to facilitate biomarker detection at the cellular level. These works mostly apply several functionalization chemistries to conjugate cell-capture “probes” such as antibodies or aptamers, using the functional groups present on the surface of GO. For example, Yoon et al. employed GO nanosheets in a microfluidic chip for capturing cells (Figure 8A) [150]. Herein, GO nanosheets, functionalized with polyethylene glycol (PEG), are directly attached on to a patterned gold surface through self-assembly by using a positively charged intercalating agent [151]. They then modified the GO surface with NeutrAvidin to immobilize the biotinylated epithelial-cell adhesion molecule antibody (anti-EpCAM) for capturing circulating tumor cells (CTCs) from the blood sample within the fluid channel (Figure 8B). They showed high sensitivity at low concentration of target cells (73% ± 32.4 at 3–5 cells/mL blood), as shown in Figure 8C, showing capture of cells at the edges of the flower pattern (Figure 8D). This system was later developed to combine the advantages of the biocompatible GO interface with a thermoresponsive polymer that promotes effective cell release for subsequent analysis by lowering the system’s temperature [152]. Released CTCs then underwent downstream analysis such as fluorescence in situ hybridization (FISH), molecular analysis, and single cell analysis, and were examined to be viable and structurally intact.

**Figure 9:**
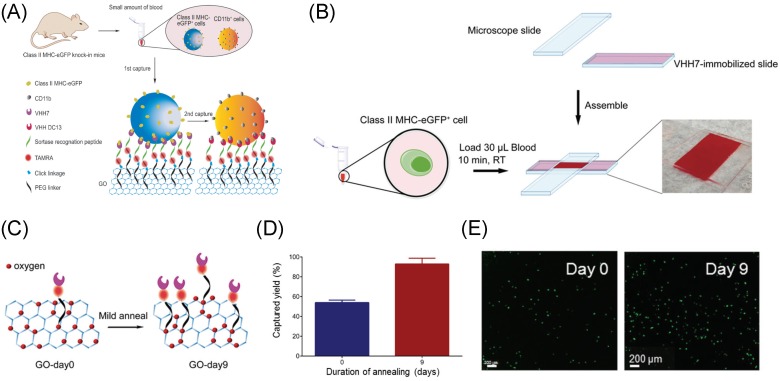
(**A**) Schematic of VHH7 and VHH DC13-based GO substrates for capture of Class II MHC-eGFP+ and CD11b+ cells from whole blood. (**B**) Schematic of the cell capture device made from glass slides, the assay conditions, and a digital photograph of the constructed and loaded capture chamber. (**C**) Schematic showing enhanced functionalization of GO nanosheets because of oxygen clustering on the graphene basal plane. GO-Day 0 represents as-synthesized GO sheets with no annealing treatment, while GO-Day 9 represents GO substrates annealed for 9 days at 80 °C to induce oxygen clustering. (**D**) Cell capture efficiency of annealed GO structure (Day 9) compared to that of as-synthesized structure (day 0). (**E**) Quantification of captured class II MHC-positive eGFP+ cells from murine whole blood samples comparing Day 0 and Day 9 samples. Figures reproduced with permission from: (**A**) [153], Wiley^©^; and (**B**–**E**) [35], ACS^©^.

In 2015, a device which integrated GO nanosheets with single domain antibodies was developed for selective capture of Class II MHC-positive (MHC+) and CD11b-positive (CD11b+) cells from small volumes (≈30 μL) of peripheral blood with minimal handling in a device of simple geometry [153]. These single-domain antigen-binding fragments were derived from camelid heavy-chain-only antibodies, known as VHHs or nanobodies, and were directly conjugated with sortase in a uniform orientation onto GO nanosubstrates (Figure 9A). This work was then extended to fully demonstrate the tunability of material properties by adjusting the sp${}^{2}$-to-sp${}^{3}$ ratios via simple mild annealing of GO (Figure 9B) [35]. The heating process induced oxygen clustering on the graphene basal plane, which resulted in enhanced chemical functionalization of GO (Figure 9C). By simply engineering the structural composition and chemistry of GO, rather than modifying the device architecture, this treated-GO platform provided an efficiency of ∼92% for capturing Class II MHC-positive cells from murine whole blood at room temperature (Figure 9D,E). In addition, to enhance the biocompatibility of GO surface functionalization or coatings, both studies used PEG-linkage to minimize system cytotoxicity [154].

Another publication has illustrated the ability of GO in multiplex detection by using aptamer-conjugated GO membranes for capturing and identifying multiple types of CTCs, including SKBR3 breast cancer cells, LNCaP prostate cancer cells, and SW-948 colon cancer cells [155]. Multiple surface markers, including S6 aptamers that bind specifically to HER2, A9 aptamers for PSMA binding, and the YJ-1 aptamers for CEA specific binding, were modified and covalently attached to 20–40 μm porous GO membranes. These GO-membranes could capture CTCs selectively and simultaneously from infected blood, with a capture efficiency as high as 98%. This work utilized amine-functionalized PEG as a cross-linking agent to build the GO foam with 3D porous architecture by interconnecting the -COOH group on GO sheets via the -NH2 amine groups of PEG. Cells were incubated on the membrane for 24 h, and cell viability of 97% was observed. All the cytotoxicity results show excellent biocompatibility of the membrane.

The above studies have illustrated that GO can serve as a foundational nano-bio interface in rare cell isolation, detection and characterization devices. By integrating physical and chemical properties of this biocompatible nanomaterial into cellular-level research, not only can the sensitivity and specificity be enhanced, but also the human-involved sample pre-processing or complex system architecture can be reduced.

**Figure 10:**
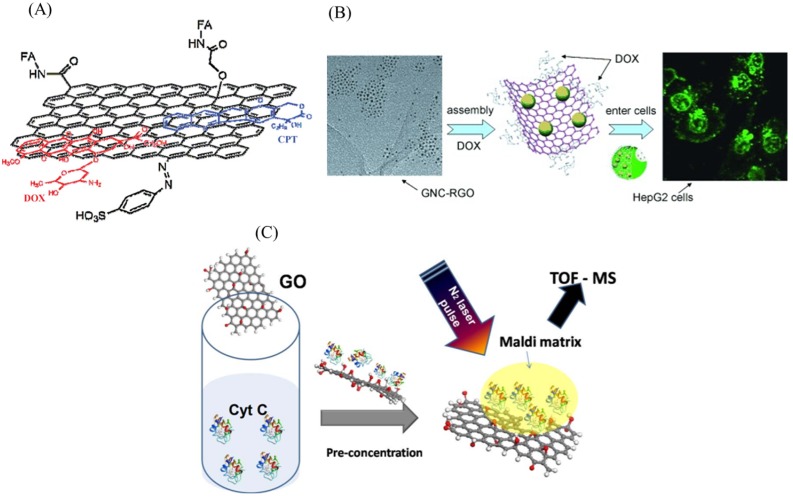
(**A**) Schematic representing the loading of DOX and CPT drugs onto FA-NGO on both sides of the graphene sheets. (**B**) Schematic of gold nanoclusters (GNCs) impregnated onto rGO nanosheets as nanocarrier for doxorubicin (DOX) for the inhibition of HepG2 hepatocarcinoma cells. (**C**) Schematic of GO platform for pre-concentration of Cyt C coupled with MALDI-TOF MS for the detection of Cytochrome C protein solution. Figures reproduced with permission from: (**A**) [159], Wiley^©^; (**B**) [160], Wiley online library^©^; and (**C**) [163], ACS^©^.

The ultrahigh surface area (2630 m${}^{2}$/g) and the *sp*${}^{2}$-hybridized carbon area offered by graphene make them superior to other nanomaterials for loading large amount of drug molecules. Hence, they are sought for drug-delivery applications. They have been used as nanocarriers for anti-cancer therapy where the ligand targeting the cancer cells can be efficiently attached on to GO [156]. Furthermore, anti-cancer drugs can also be directly attached on to the surface of graphene and GO for cancer therapy. For instance, Doxorubicin and SN38 anti-cancer drugs were directly loaded on nano-GO through simple physisorption [157,158]. To ensure the selectivity against cancer cells, CD20+ antigen was also co-immobilized. Zhang et al. studied the anticancer activity of the mixed drugs DOX and camptothecin (CPT) onto the folic acid attached GO (Figure 10A) against MCF-7 breast cancer cells [159]. They found that loading two drugs was better cytotoxic than loading a single drug. Wang et al. used GO-gold nanoparticle composite (Figure 10B) to enhance the anti-cancer activity and found that DOX loaded nanocomposite inhibits HepG2 cell growth more efficiently [160]. In addition to drug delivery, GO composite can also be used for gene delivery application. In a study, polyethylenimine (PEI)-functionalized GO was used to load small interference RNA (siRNA) for inhibiting protein expression by selective cleavage of messenger RNA [161]. Similarly, chitosan-functionalized GO (CS-GO) complex was used as a novel nanocarrier to load water insoluble anticancer drug CPT. They demonstrated that this composite has superior loading and high cytotoxicity towards HepG2 and HeLa cell lines [162].

Due to high heat dissipation and electron transfer properties, graphene has been considered as an effective matrix in laser desorption ionization mass spectrometry (LDI MS) applications in which graphene can absorb and transfer UV laser energy to the molecules efficiently for their detection. Using this technique, aromatic pollutant molecules, ssDNA and proteins have been detected using graphene as an affinity probe [163,164,165]. Figure 10C shows a schematic of the detection of low concentration of cytochrome c.

## 4. Outlook

Excellent electrochemical properties of graphene and GO-based nanomaterials and nanocomposites offer great opportunities to develop electrochemical biosensors and immunosensors for the detection of clinically important biomarkers. Moreover, these nanomaterials are promising for drug and gene delivery applications, especially for the treatment of cancer.

Graphene has also started making its way toward commercial applications. Nokia has a patent on a graphene-based photodetector in which graphene is used as a photon-collecting layer. These photodetectors are claimed to be cheaper than commercial photodetectors [166]. Nokia has also patented a graphene-based flexible photon battery, a self-charging battery that can be printed on flexible substrates. Recently, Samsung Electronics Co. Ltd developed a nano graphene-enhanced lithium-ion battery in which graphene acts as an active anode material exhibiting a stable charge and discharge cycling response, a high specific capacity per unit mass, a high first-cycle efficiency, a high capacity per electrode volume, and a long cycle life [167]. In 2015, a Korean based research institute called Electronics and Telecommunications Research Institute (ETRI) successfully developed a highly sensitive, flexible, and washable textile type gas sensor using graphene. A wearable cloth with these gas sensors can check the air condition and could be useful for firefighters. Flexible and transparent graphene-based sensors were developed that can be attached to the skin to detect biosignals. Researchers from Trinity college, Ireland developed a wearable sensor that can detect pulses and breath based on electric conductivity [168]. These type of sensors can also be used in automobile industry. Similarly, a sensor for detecting airborne chemicals released from exhaled breath or skin was developed by a research team at the University of Michigan.

Even though graphene-based nanomaterials are vastly studied for biomedical applications, certain challenges still exist in this field. For instance, controlling the biomolecule orientation and function on the graphene surface are still challenges and they limit the performance of the biomedical devices. To overcome this limitation, synthesis of uniform graphene is highly essential to improve the repeatability and accuracy of the biomolecule detection. It opens future directions to develop new fabrication methods. Similarly, mass production of graphene quantum dots with high yield is required for bioimaging applications. Moreover, it is also very important to study the long-term toxicity of graphene nanomaterials and the cellular uptake mechanism.

Although GO has an abundance of oxygen functional groups on its surface, effective utilization of these functional groups has remained difficult owing to their amorphous nature. Effective synthesis protocols to prepare GO structures with a particular oxygen functional group or developing ways to control the chemical structure of GO would be an interesting direction to pursue in the future. Selectively functionalizing a specific functional group over another in a controllable manner would enable multi-functionalization of GO. To further tailor graphene-based biosensing systems for clinical use and, to obtain consistent results, researchers have to develop scalable methods for immobilizing different biomolecules to achieve one-step, no-wash multiplex detection. Overall, graphene-based nanomaterials emerge as promising platform for biomedical applications with a continuing need for effective collaboration among different scientific communities such as chemistry, physics, biology and medicine to enable its applicability.

## Figures and Tables

**Table 1:** Performance of enzymatic biosensors based on graphene based nanomaterials for the detection of clinically important biomarkers.

Sensing Electrode	Detected Element	Detection Range	Detection Limit	Reference
P-L-His–rGO	H${}_{2}$O${}_{2}$	0.2 μM to 5 mM	0.05 μM	[59]
rGO	H${}_{2}$O${}_{2}$	1.5–28.5 μM	0.5 μM	[58]
CeO${}_{2}$–rGO	H${}_{2}$O${}_{2}$	0.1–500 μM	0.021 μM	[60]
rGO–ZnO	Glucose	0.2–6.6 mM	0.2 mM	[68]
rGO–AgNPs	Glucose	0.5–12.5 mM	0.16 mM	[89]
AuNPs–GR–CNTs	Glucose	10 mM to 2 mM	4.1 mM	[90]
rGO–cyclodextrin	Glucose	50 μM to 3.0 mM	59.74 mM	[91]
PANI-modifed SnO${}_{2}$–rGO	Glucose	0.1 nM to 5 mM	0.26 nM	[92]
Fe${}_{3}$O${}_{4}$–rGO	Glucose	50 μM to 1 mM	0.1 μM	[69]
rGO–Fullerene–C60	Glucose	100 uM to 12.5 mM	35 μM	[93]
ZrO${}_{2}$–rGO	Glucose	14–290 μM	130 μM	[70]
AuNPs–ErGO–PAH	NADH	0.01 to 5 mM	3.5 μM	[94]
Au–AgNPs–P(L-Cys)–ErGO	NADH	0.017 to 1.84 μM	5 μM	[95]
rGO	NADH	0–500 μM	0.6 μM	[96]
Chitosan–GO	DNA	10 fM to 50 nM	10 fM	[97]
GR–ErGO	DNA	10 pM to 0.1 μM	0.15 fM	[98]
AuNPs–rGO	DNA	0.1 fM to 0.1 μM	35 aM	[99]
PPy–grGO	Cholesterol	0.01 to 6 mM	3.78 μM	[84]
Chitosan–GR	Cholesterol	0.005 to 1 mM	17.39 μM	[100]
PtNPs–GR	Cholesterol	0.035 to 12 mM	0.2 μM	[86]
Pd-Pt NPs–GR	Cholesterol	2.2 μM to 0.52 mM	0.75 mM	[101]
GR–PVP–PANI	Cholesterol	50 μM to 10 mM	1 μM	[102]
rGO–dendritic Pd	Cholesterol	0.005–0.014 mM	0.05 μM	[85]
CS–GR	Cholesterol	0.005–1.0 mM	0.715 μM	[100]
TiO${}_{2}$ nanowires–3D GR	Cholesterol	0.05–8.0 mM	6 μM	[103]

**Table 2:** Performance of non-enzymatic biosensors based on graphene based nanomaterials.

Sensing Electrode	Detected Element	Detection Range	Detection Limit	Reference
PtNPs–MnO${}_{2}$–rGO	H${}_{2}$O${}_{2}$	2 μM to 133 mM	1 μM	[107]
rGO–tyrosine	H${}_{2}$O${}_{2}$	0.1–2.1 mM	80 μM	[108]
Ni(OH)${}_{2}$–rGO–MWCNTs	H${}_{2}$O${}_{2}$	10–9050 μM	4 μM	[109]
rGO–nPPY	H${}_{2}$O${}_{2}$	1–4 μM	34 nM	[110]
GR–PtNiNPs	Glucose	0.5–35 mM	10 μM	[111]
GR–CuO NPs	Glucose	1 μM to 8 mM	1 μM	[112]
GO–CuONPs	Glucose	2.79 μM to 2.03 mM	0.69 μM	[113]
rGO–Ni(OH)${}_{2}$	Glucose	15 μM to 30 mM	15 μM	[114]
rGO–Au-CuO NPs	Glucose	1 μM to 12 mM	0.01 μM	[115]
N-rGO–Mn${}_{3}$O${}_{4}$ NPs	Glucose	1.0–329.5 μM	0.5 μM	[116]
rGO–Pt–NiO	Glucose	0.008–14.5 mM	2.67 μM	[117]
AgPt–rGO	Glucose	0.003–7.72 mM	1.8 μM	[118]
NiO–CVD-GR	Cholesterol	2–40 μM	0.13 μM	[119]
PANInf–GMF	Cholesterol	1.93 to 464.04 mg dL${}^{-1}$	1.93 mg dL${}^{-1}$	[120]
GR–β-CD	Cholesterol	1–100 μM	1 μM	[121]
GO–MIP	Cholesterol	0.1 M–1 nM	0.1 nM	[122]

**Table 3:** Performance of immunosensors based on graphene-based nanomaterials.

Sensing Electrode	Detected Element	Detection Range	Detection Limit	Reference
GR–CS–AuNPs	CEA	0.5–60 ng mL${}^{-1}$	0.1 ng mL${}^{-1}$	[130]
GR–MWCNTs–CS–AuNPs	EBNA-1	0.05–6.4 ng mL${}^{-1}$	0.7 pg·mL${}^{-1}$	[131]
GR–AuNPs	PSA	0–10 ng mL${}^{-1}$	0.59 ng mL${}^{-1}$	[132]
GR–TiO${}_{2}$	ErbB2	1 fM–0.1 μM	0.06 ng mL${}^{-1}$	[133]
PtCu@rGO–g-C${}_{3}$N${}_{4}$	PSA	50 fg mL${}^{-1}$–40 ng mL${}^{-1}$	16.6 fg mL${}^{-1}$	[134]
AuNPs–GO	ErbB2	0.37–10 nM mL${}^{-1}$	0.16 nM	[123]
S-doped GR–PANI	CEA	0.1 pg·mL${}^{-1}$ to 0.3 ng mL${}^{-1}$	30 fg mL${}^{-1}$	[135]
CdS QDs@PS–GO–PANI	K562 cells	10${}^{-1}$ ×10${}^{-7}$ cells per mL	3 cells per mL	[124]
Hemin-GR–PdNPs	PSA	0.025–205 ng mL${}^{-1}$	8 pg·mL${}^{-1}$	[136]
PPy–ErGO	BRCA1 gene	10 fM to 0.1 μM	3fM	[137]
